# Sublingual microcirculation: a case report

**DOI:** 10.1186/s13256-019-2118-4

**Published:** 2019-06-12

**Authors:** Jonas D. Scheuzger, Anna Zehnder, Desirée Yeginsoy, Martin Siegemund

**Affiliations:** Department for Anesthesia, Surgical Intensive Care, Prehospital Emergency Medicine and Pain Therapy, University Hospital Basel, Spitalstrasse 21, CH-4031 Basel, Switzerland

**Keywords:** Microcirculation/physiology, Monitoring, physiologic/methods, Colectomy, Sepsis, Critical illness, Intensive care units

## Abstract

**Introduction:**

Sublingual microcirculation monitoring is suitable for bedside use in critically ill patients. We present a case in which severely impaired sublingual microcirculation was the first alarming sign of an early deterioration of the patient’s medical situation.

**Case presentation:**

This is the case of a 58-year-old white woman admitted to our intensive care unit after the removal of parts of her small intestine due to a volvulus. Her microcirculation was checked the day after surgery in terms of an ongoing study and predicted a massive deterioration of her clinical situation.

**Conclusions:**

This case highlights the potential value of monitoring the microcirculation in critically ill patients. Two full hours could have been saved for diagnostic workup and earlier treatment had we considered the impaired microcirculation alone as a warning sign. Regardless of the supposed cause, impaired microcirculation should alert the responsible physician and should be followed by a diagnostic workup. Sublingual microcirculation monitoring can be useful in intensive care units to detect a deteriorated microcirculation earlier than with standard monitoring.

**Electronic supplementary material:**

The online version of this article (https://doi.org/10.1186/s13256-019-2118-4) contains supplementary material, which is available to authorized users.

## Introduction

Hemodynamic and laboratory parameters are monitored continuously in intensive care patients. Although an intact microcirculation is the key player in tissue metabolism, evaluating microcirculation is not part of daily routine in the intensive care unit (ICU). Peripheral oxygen saturation and serum lactate only provide indirect evidence of the microcirculatory system. Impaired microcirculation results in attenuated metabolic blood supply, which can cause cell damage. More importantly, microcirculatory flow (MCF) can react independently of global circulatory parameters (for example, blood pressure, vasopressors) [1, 2].

Sublingual incident dark field (IDF) imaging provides physiological insight into the microcirculation in real time. The CytoCam-IDF (Braedius Medical, Huizen, the Netherlands) is a handheld device available for bedside use [3]. The CytoCam records videos non-invasively through the mucosa (for example, in the sublingual region). This provides us with the possibility to observe the microcirculation in critically ill patients.

The reasons for an impaired microcirculation are not yet fully understood. Intact microcirculation depends on blood cells, hemoglobin, coagulation factors, intravascular fluid status, vasoactive drugs, and tissue-specific variables. An impaired MCF may even be the very first sign of alarm for a deteriorating situation in critically ill patients. Canaries were used in nineteenth century coal mines to alert miners to elevated levels of carbon monoxide [4]. Monitoring microcirculation may function in a similar way. We report a case of a severely altered microcirculatory perfusion in the presence of completely normal circulatory and laboratory parameters.

## Methods

**Fig. 1:**
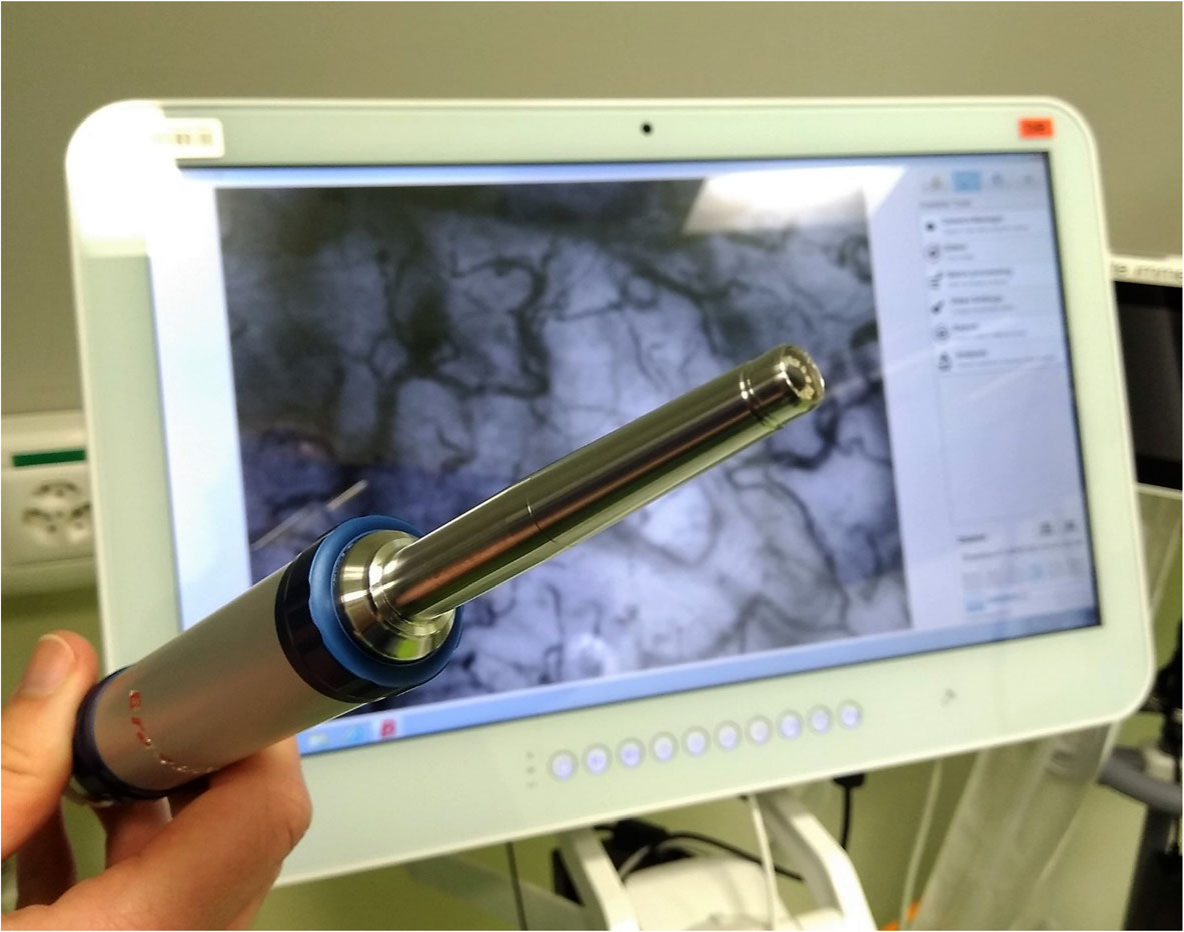
CytoCam device at our intensive care unit

We regularly perform CytoCam measurements in critically ill patients as a part of ongoing studies in our department. We used the latest third-generation handheld microscope CytoCam (Braedius, Huizen, Netherlands). This handheld microscope currently provides the best optical resolution compared to other devices and is suitable for bedside use [5]. The device consists of a pen-like probe incorporating IDF illumination, a principle originally introduced by Sherman *et al.* (Fig. 1) [6]. In this case, we took five videos and used the three best records according to the microcirculation image quality score (MIQS) [7] for offline analysis. The careful application of the tip-like microscope in the sublingual fold avoids pressure artefacts. We used the microvascular flow index (MFI) and De Backer's Score [8] to describe semi-quantitative parameters like total vessel density (TVD), proportion of perfused vessels (PPV), and perfused vessel density (PVD). The operator attended a training course, and two independent observers performed offline analyses.

## Case presentation

**Fig. 2:**
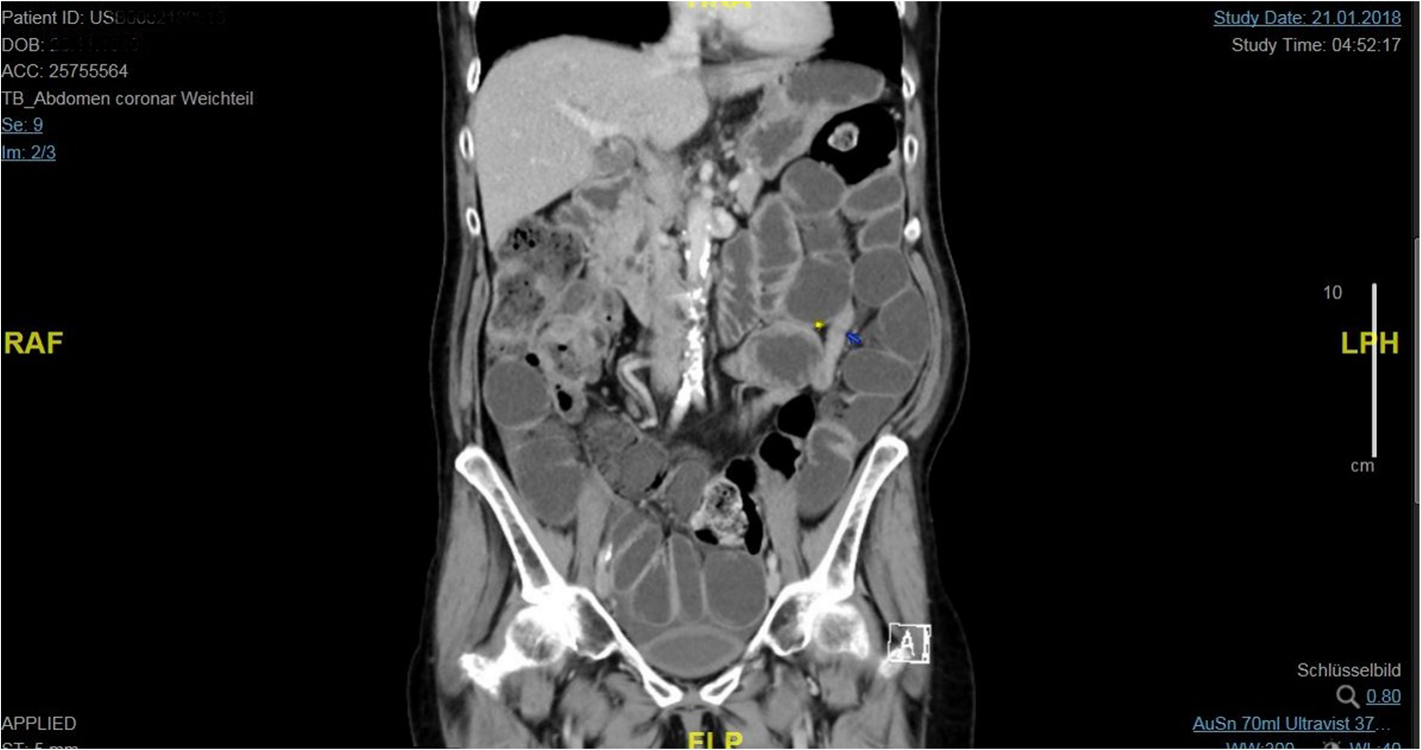
Frontal with triple contrast shows a distended small intestine in the left upper abdomen. Caliber jump of the intestine is marked with a *yellow* and *blue do*t

A 58-year-old white woman, weighing 55 kg and with a body mass index of 23, was admitted to our hospital suffering from general weakness, coughing with purulent sputum, fever, and nausea. She presented in a stable general condition, except for schizophrenia with mild cognitive impairment. Prior to admission, she received clozapine (250 mg/day) for schizophrenia. She had no other relevant diagnoses and interventions in her past medical history. She lived in an assisted living facility and was employed in a protected workplace program. She was able to take care of herself and was able to perform daily tasks on her own (for example, grocery shopping). She has no direct relatives and was raised in a children’s home. On admission, she was awake with a Glasgow Coma Scale (GCS) of 15 and was temporal, local, and autopsychic oriented. She showed no neurological deficiency. She was hemodynamically stable with heart rate of 100 beats per minute (bpm), blood pressure of 99/70 mmHg, respiratory rate of 14/minute, and body temperature of 38.9 °C. She had signs of mild dyspnea, coughing, and wheezing at auscultation. Her abdomen was soft without tenderness on palpation. Bowel sounds were equally present. An influenza screening test was negative. Urinary and blood cultures showed no bacterial infection. Blood samples on admission showed an elevated C-reactive protein (CRP) of 39 mg/L, leukocytes of 9.9 g/L, and a lactate level of 2.1 mmol/L. Creatinine clearance, liver function, electrolytes, and counted blood cells were all within normal ranges. With a tobacco smoking history of 40 pack-years, she was now treated for exacerbated chronic obstructive pulmonary disease and antibiotic therapy with intravenously administered amoxicillin/clavulanic acid (1.2 g three times a day). Prednisone (50 mg/day) was also started. Two days after admission, she suffered acute vomiting and presented a diffuse pressure-resistant and distended abdomen. Abdominal computed tomography showed a volvulus in the small intestine, 20 cm from the ileocecal valve, confirming the indication for emergency laparotomy (Fig. 2).

After surgery, she arrived in the ICU under sedation with propofol (80 mg/hour) and fentanyl (0.05 mg/hour), decreased bowel sounds, and normal cardiopulmonary parameters. Initially, she received 0.5 mg haloperidol as prophylaxis for delirium. She was continuously administered noradrenaline (0.34 μg/kg per minute) to maintain circulation and Ringer’s acetate for volume support. We treated her respiratory infection with piperacillin/tazobactam (13.5 g/day). For volume support, she received 5000 ml Ringer’s acetate during the first 24 hours. Her urinary output was supported with furosemide (35 mg) over the first 24 hours. In addition, she received anticoagulation with dalteparin (5000 IU).

The initial period was uneventful. Although ventilator weaning was intended for our patient and sedation was reduced, she remained dependent on positive end-expiratory pressure and extubating was not possible. Under volume therapy and a positive fluid balance of 4 liters the next morning, noradrenaline could be partially reduced and maintained at 0.25 μg/kg per minute. Her first post-surgery blood check at 5 p.m. showed a CRP of 179 mg/L, leukocyte count of 17 × 10^9^/L, hemoglobin at 81 g/L, creatinine at 118 μmol/L, and serum lactate at 1.5 mmol/L. She was slightly agitated and received a total 4.5 mg haloperidol over 24 hours for delirium treatment.

**Fig. 3:**
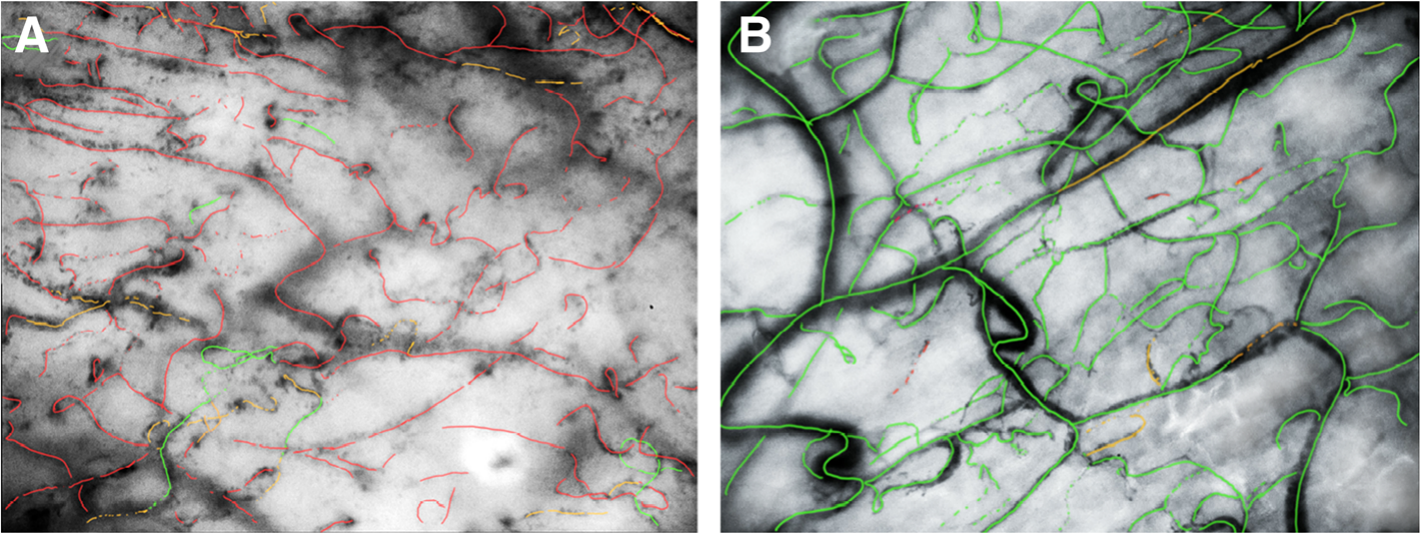
**a**. Impaired microcirculation represented by the case described (recording 1) versus **b** normal microcirculation. The images were extracted from the video recording and colorized according to the original video presentation for a better visualization with: *red* = *no flow*, *orange* = *intermittent flow*, *green = normal flow*

In the morning, we included her in a study and routinely measured her microcirculation. The microcirculatory measurement revealed an almost absent MCF (Fig. 3, see also Supplementary content – Additional file 1: Video S1, *Impaired sublingual microcirculation of the described case,* in comparison with Additional file 2: Video S2*, intact microcirculation of a member of the study team*). In addition, offline analysis showed an MFI of far below normal (2.6 is the accepted threshold) for all three recordings. Even in the recording with the most perfused vessels (Table 1), less than 25% of the vessels were perfused (PPV).

**Table 1:** Flow variables of the three selected recordings

Parameters	Recording 1	Recording 2	Recording 3
MFI	0.3	0.5	0.75
VD (De Backer)	9.6	6.6	8.1
PVD (De Backer)	0.49	1.11	1.98
PPV (De Backer), %	5.33	16.7	24.2
MIQS	1	1	1

*MFI* microcirculatory flow index, *MIQS* microcirculatory image quality score, *PPV* proportion of perfused vessels, *PVD* perfused vessel density, *VD* vessel density

Except for the altered microcirculation, circulatory and laboratory parameters showed no clear indication for a deterioration of the medical situation. Post-surgical CRP and leukocytes were expected to be high. Infrared blood oxygen saturation (SpO_2_) inconsistently showed low values between 50 and 90% by a fingertip detector. We initially interpreted this to be a malfunction due to a cold periphery.

Because of the normal hemodynamic measurements, we chose an expectant strategy and increased intravenous fluid administration. Arterial blood gas analysis showed only moderate rise of lactate levels from 1.5 to 3 mmol/L (Table 2). A laboratory examination 80 minutes later indicated a rapid deterioration of the situation with a lactate level of 5.4 mmol/L and a decreased hemoglobin level of 59 g/L (36.6 mmol/L).

**Table 2:** Monitoring values

Parameters	1 BS (08:20)	2 BS (10:30)	3 BS (11:50)	4 BS (12:20)
Lactate (mmol/L)	1.5	3	5.4^↑^	6.5^↑^
Hemoglobin (g/L)	82	82	59^↑^	59^↑^
pH	7.36^↓^	7.36^↓^	7.346^↓^	7.321^↓^
HCO_3_ (mmol/L)	24.7	23.8	21.1	19.9
PCO_2_ (kPa)	5.9^↑^	5.6^↑^	5.2	5.3
PO_2_ (kPa)	14.7	15.8	16.9	17.1
Anion gap (mmol/L)	7.3^↓^	7.2^↓^	11.9	14.1
Potassium (mmol/L)	4.1	4.5	4.1^↓^	5.4
Heart rate (bpm)	117	118	119	120
Mean arterial pressure (mmHg)	61	61	64	66
Bladder temperature (°C)	37.1	38.0	38.3^↑^	38.5^↑^
Noradrenaline (μg/minute)	14	13	12	11

*bpm* beats per minute, *BS* blood sample, *HCO*_*3*_ bicarbonate, *PCO*_*2*_ partial pressure of carbon dioxide, *PO*_*2*_ partial pressure of oxygen, *↑* increased value, *↓* decreased value

Transfusion of 2 units of packed cells was initiated, and an arterial blood gas sample was drawn after 30 minutes. Transthoracic ultrasound showed a hyperdynamic, underfilled left ventricle, and abdominal ultrasound gave no further information. She underwent an immediate surgical re-evaluation.

The laparotomy showed a dilated and visually ischemic descending colon down to the sigmoid. The previous ileocecal anastomosis was still intact and the ileum was vital. The entire colon was removed, and an ileostomy was implanted in her abdominal wall. A third look surgery a few days later showed an intact and vital remaining bowel. Postoperative microcirculation and blood samples returned to normal, and she recovered slowly without further complications from her severe condition. She could be discharged from our hospital 4 weeks after admission and returned to her domestic environment. In the follow-up consultation after 6 months at our hospital she refused a retrocession of the stoma. She managed the daily care of the ileostomy by herself and felt comfortable with it. Except for the permanent ileostomy, she did not have any residual symptoms from the incident and integrated herself in the daytime routine. No further consultation was planned.

## Discussion

**Fig. 4:**
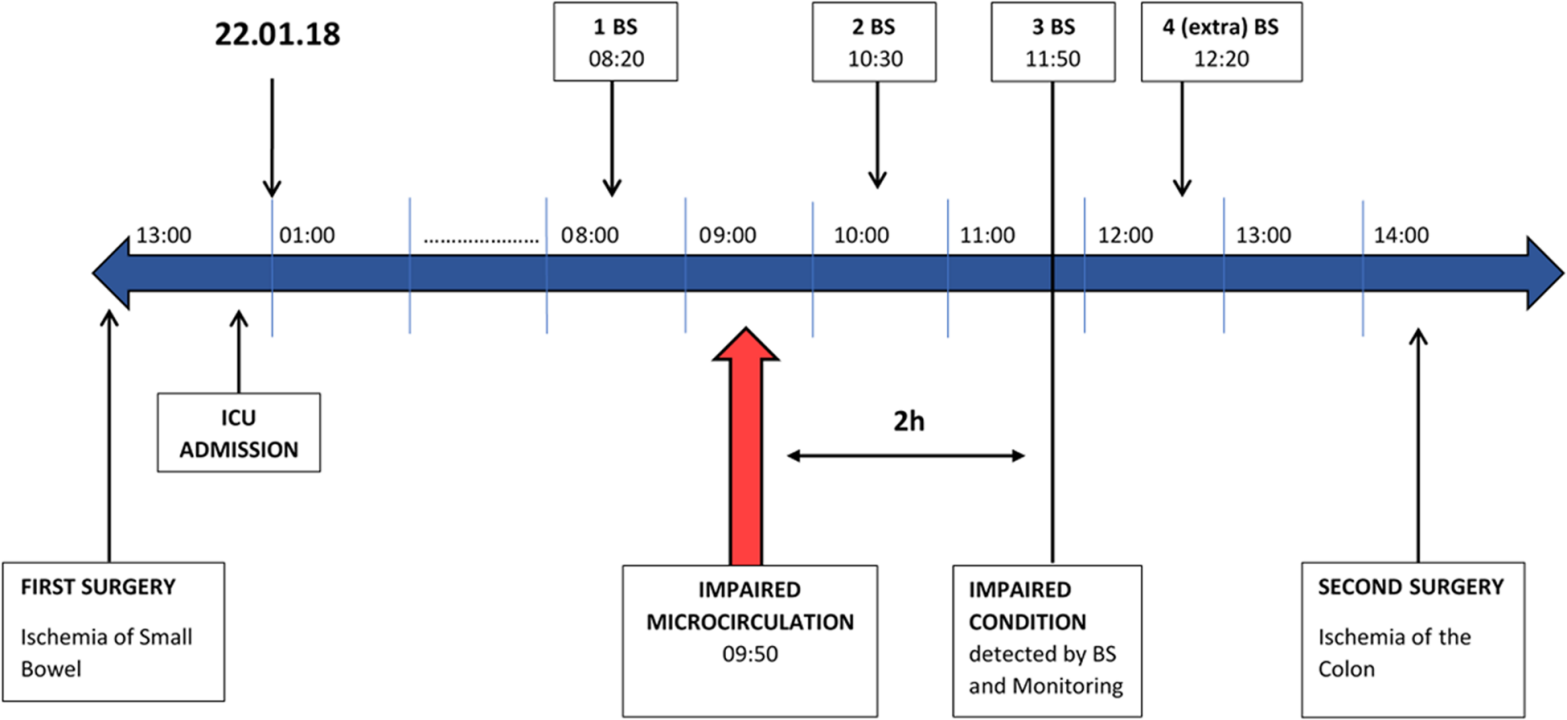
Timeline of the case since intensive care admission. *BS* blood sample, *ICU* intensive care unit

To the best of our knowledge, this is the first report of severely impaired sublingual microcirculation in a patient, clearly indicating a deterioration of the clinical situation, prior to standard hemodynamic monitoring and regular blood samples. This case demonstrates how observation of the microcirculation in a severely ill patient can act as an advanced warning compared to standard hemodynamic monitoring and regular blood samples. The alarm aspect of detecting early deterioration of the microcirculation is analogous to the old safety practice of using canaries in coal mines to serve as an early warning of an elevated level of carbon monoxide. If impaired microcirculation had been ascribed greater attention in this case, a minimum of 2 hours could have been saved for diagnostic workup (Fig. 4). However, it is unclear at which point in time the microcirculation started to deteriorate before the measurement.

Although microcirculatory measurement is not a routine diagnostic tool for any specific type of disease, an impaired microcirculation should not be overlooked. Tissue oxygenation takes place by direct exchange of oxygen from erythrocytes to the endothelial surface of small vessels and capillaries of the microcirculatory system. Previous studies in patients with sepsis [9] and cardiac [10] patients have demonstrated that persistent MCF alterations unresponsive to therapy are independently associated with adverse outcome. Despite intact macro-hemodynamic parameters (for example, blood pressure and cardiac output), tissue hypoxia due to MCF collapse or dysregulation occurs in patients with shock [1, 2].

Early detection of a massive deterioration of the MCF before standard monitoring that was able to indicate this degree of severity may be an accidental finding. Great caution is advised, and a generalization without further studies cannot be made from a case report. However, restoration of MCF is the key goal in patients with shock, and impaired MCF should at least alert the clinician in charge. Especially, in apparently stable critically ill patients (for example, with sepsis or cardiac failure), a point-in-time measurement in the ICU may help to improve patient management with therapeutic or surgical interventions and detect a critical deterioration earlier. For further study-related purposes, we suggest performing a point-in-time microcirculatory measurement once daily in ICU patients who fulfill one of the following criteria:

- patients dependent on noradrenaline (> 0.2 μg/kg per minute) for circulatory support
- patients dependent on vasopressin
- patients with a skin mottling score ≥ 2 [11].

From our experience, the measurement is feasible and can be performed by a trained observer at the bedside in less than 5 minutes.

Microcirculation was severely impaired sublingually, even more so in the colon tissue. Although there is no common vascular supply to the tongue and descending colon, we assume that if the sublingual microcirculatory system, which is directly supplied by the carotid arteries and therefore in close proximity to the heart, shows impaired MCF, then capillary regions more distant to the heart are likely to be affected too.

The described case leaves the exact mechanism that led to the altered microcirculation unclear. Data suggest that various mechanisms are involved in the development of impaired microcirculation. Reduced glycocalyx in sepsis promotes adhesion and rolling of leukocytes to the endothelium and reduces blood flow. Increased levels of endotoxins impair backward communication through perivascular nerves to upstream arterioles. Further, endothelial dysfunction with increased capillary leak may lead to relative hypovolemia and altered MCF [12].

Independent from the causative mechanism, observation of the microcirculation in apparently stable ICU patients may help to adjust the therapy or extend diagnostic workup before definitive hemodynamic deterioration.

## Conclusion

In an individual patient, impaired MCF should be taken as a serious warning sign for an imminent deterioration, like a silent canary was for old-time coal miners.

Further research should be conducted to evaluate the value of microvascular blood flow as routine monitoring for apparently stable patients.

## Additional files

Additional file 1: **Video S1.** Impaired sublingual microcirculation of the described case (recording 1). (MP4 7247 kb)

Additional file 2: **Video S2.** Intact sublingual microcirculation of a member of the study team. (MP4 7614 kb)

## Abbreviations

bpm: Beats per minute
CRP: C-reactive protein
GCS: Glasgow Coma Scale
ICU: Intensive care unit
IDF: Incident dark field
MCF: Microcirculatory flow
MFI: Microvascular flow index
MIQS: Microcirculation image quality score
PPV: Proportion of perfused vessels
PVD: Perfused vessel density
SpO_2_: Blood oxygen saturation
TVD: Total vessel density
